# Whole-genome resequencing of a global collection of Napier grass (*Cenchrus purpureus*) to explore global population structure and QTL governing yield and feed quality traits

**DOI:** 10.1093/g3journal/jkaf113

**Published:** 2025-05-23

**Authors:** Abel Teshome, Hailu Lire, Janet Higgins, Temesgen Magule Olango, Ermias Haile Habte, Alemayehu Teressa Negawo, Meki Shehabu Muktar, Yilikal Assefa, Jorge Fernando Pereira, Ana Luisa Sousa Azevedo, Juarez Campolina Machado, Desterio Nyamongo, Jiyu Zhang, Yan Qi, William Anderson, Jose De Vega, Christopher Stephen Jones

**Affiliations:** Feed and Forage Development, International Livestock Research Institute, P.O. Box 5689, Addis Ababa, Ethiopia; Ethiopian Institute of Agricultural Research, Wondogenet Agricultural Research Centre, P.O. Box 2003, Wondogenet, Ethiopia; Earlham Institute, Norwich Research Park, Norwich NR4 7UZ, UK; Almaviva, Via di Casal Boccone, 188-190, Rome 00137, Italy; Feed and Forage Development, International Livestock Research Institute, P.O. Box 5689, Addis Ababa, Ethiopia; Feed and Forage Development, International Livestock Research Institute, P.O. Box 5689, Addis Ababa, Ethiopia; Feed and Forage Development, International Livestock Research Institute, P.O. Box 5689, Addis Ababa, Ethiopia; Feed and Forage Development, International Livestock Research Institute, P.O. Box 5689, Addis Ababa, Ethiopia; Embrapa Dairy Cattle, Juiz de Fora 36038-330, Brazil; Embrapa Dairy Cattle, Juiz de Fora 36038-330, Brazil; Embrapa Dairy Cattle, Juiz de Fora 36038-330, Brazil; Kenya Agricultural and Livestock Research Organization, P.O. Box 57811, Nairobi 00200, Kenya; State Key Laboratory of Grassland Agro-Ecosystems, College of Pastoral Agriculture Science and Technology, Lanzhou University, Lanzhou 730020, China; State Key Laboratory of Grassland Agro-Ecosystems, College of Pastoral Agriculture Science and Technology, Lanzhou University, Lanzhou 730020, China; Crop Genetics and Breeding Research Unit, USDA-ARS, 115 Coastal Ways, Tifton, GA 31793, USA, retired; Earlham Institute, Norwich Research Park, Norwich NR4 7UZ, UK; Feed and Forage Development, International Livestock Research Institute, Box 30709, Nairobi 00100, Kenya

**Keywords:** agronomic traits, drought, GWAS, elephant grass, *Pennisetum purpureum*, nutritional traits, QTL, SNP markers, underutilized crop, plant genetics and genomics

## Abstract

Napier grass (*Cenchrus purpureus*) is a C_4_ perennial grass species native to Sub-Saharan Africa and widely used as livestock feed in the region. In this study, we sequenced the genomes of 450 Napier grass individuals from 18 countries, identifying over 170 million DNA variants (SNPs and Indels). Approximately 1% of these SNPs were informative and used to assess genetic diversity within the collection. Our resequencing study provided valuable insights into the global genetic diversity of Napier grass. Additionally, a genome-wide association study on 2 independent populations identified multiple quantitative trait loci significantly associated with key agronomic traits, including biomass yield, nitrogen and cellulose content. These findings serve as a crucial resource for preserving and understanding Napier grass genetic diversity in the context of climate change. Moreover, they will support genomics-based breeding programs aimed at developing high-yielding and drought-tolerant varieties for forage and biofuel production.

## Teaser

Napier grass (*Cenchrus purpureus*) is a perennial grass species native to Sub-Saharan Africa (SSA), primarily used to feed cattle in SSA. The authors’ resequencing study provided valuable insights into the genetic diversity across a global Napier grass collection. Furthermore, a genome-wide association study on two independent populations, identified multiple quantitative trait loci (QTL) that were significantly associated with desirable agronomic traits. Therefore, their results will serve as a valuable resource to spearhead genomics-based breeding programs to develop high-yielding and drought-tolerant varieties.

## Introduction

Globally, grasslands cover 26% of the land area, 70% of agricultural land, and play an important role as livestock feed, particularly in Sub-Saharan Africa (SSA) (FAO 2010). In SSA, popular grasses include *Cenchrus*, *Urochloa*, and *Megathyrsus* species and these grasses are critically important for smallholders and frequently used by women to maintain the livestock production systems (Njuki and Sanginga 2013; Simeão *et al*. 2021). Unfortunately, annual milk and meat production in SSA remains low compared to the global average (Balehegn *et al*. 2021). One of the significant reasons behind the below-par productivity of the livestock industry is the inadequate access to quality feeds and forages, worsened recently by the risks associated with climate change (Balehegn *et al*. 2020; Paul *et al*. 2020). Most small-scale livestock farmers in SSA rely heavily on natural common grazing lands as their primary source of forage and feed supply, mainly available during the rainy seasons (Hanan and Kahiu, 2016). Unfortunately, such grazing lands are dwindling because of the inevitable population increase, climate change, and more land being allocated for food crops (Tolera 2007; Smith *et al*. 2013; Enahoro *et al*. 2019). Consequently, livestock farmers are now more in need of productive, high quality and resilient forage varieties to support their livestock.

Napier grass or Elephant grass [*Cenchrus purpureus* (Schumach.) Morrone syn. *Pennisetum purpureum* Schumach.] is a crucial traditional forage species in SSA, growing mainly up to 2,000 m above sea level in the tropics (Habte *et al*. 2020; Mkutche 2020). It is primarily used to feed cattle in cut and carry feeding systems in Ethiopia, Kenya, Uganda, Tanzania, and Nigeria (Mwendia *et al*. 2006), because of its low cost of production, year-round availability under limited irrigation, and some degree of resilience against drought (Habte *et al*. 2020; Muktar *et al*. 2022). Due to its high biomass yield and desirable nutritional traits, Napier grass has recently garnered interest as a candidate for bio-based products and biofuels in tropical and semitropical regions of the world, such as the USA and Brazil (Anderson *et al*. 2008; Rocha *et al*. 2019; Sawasdee and Pisutpaisal 2021). Once established in the main production field, Napier grass can grow and be maintained for decades under good management practices, yielding up to 50 tons of dry matter (DM) ha^−1^ per year (Habte *et al*. 2020; Dokbua *et al*. 2021). Because of its adaptability, persistence, and versatility, it has been naturalized to Central and South America, the tropical parts of Asia, Australia, the Middle East, and the Pacific Islands (Fukagawa and Isshi 2018).

Napier grass has the potential to be included in the mainstream feed chain, particularly in the tropics, if research is focused on this species, it can also contribute to energy requirements for current and future generations. Unfortunately, Napier grass remains an underutilized crop with limited genetic and genomic tools developed to date and few cultivars available for farmers. The first reference genome was reported in 2020 (Yan *et al*. 2021) and a second, improved 1 was reported in 2022 (Zhang *et al*. 2022). The availability of these reference genomes facilitates the generation of molecular markers by elucidating their genomic positions. Here, we report on a species-level whole-genome sequencing (WGS) study of a global collection of 450 genotypes. We analyzed the collection's diversity to explore how breeding, selection, and environmental pressures have shaped the Napier grass genome in the international collections. We also analyzed the genomic regions associated with important agronomic traits, such as fresh biomass yield and plant height, and nutritional feed-quality traits, such as crude protein content. Thus, the genomic tools developed in this study will enable forage breeders to apply advanced plant breeding procedures such as genomic selection and marker-assisted breeding, which have been lacking to date for Napier grass. Furthermore, new perspectives from the study should benefit conservation efforts worldwide.

## Materials and methods

### Napier grass field evaluation

Phenotype data were assessed from 2 collections of Napier grass genotypes which were independently evaluated in this study. The first collection consisted of 84 genotypes conserved at the International Livestock Research Institute (ILRI) genebank which was evaluated in Bishoftu, Ethiopia for 2 consecutive years, in a P-rep design, replicated twice. Details of the field evaluation have previously been reported (Habte *et al*. 2020; Muktar *et al*. 2022). Briefly, 84 genotypes (72 unique and 12 check genotypes) were arranged into 4 blocks with 2 replicates. The 12 check genotypes were duplicated in each block, while the remaining 72 genotypes appeared only once. The first 2 blocks (first replicate) were subjected to a volumetric soil water content (VWC) of approximately 20%, referred to as moderate water stress (MWS). In contrast, the other 2 blocks received less water, resulting in a VWC of about 10%, referred to as severe water stress (SWS). The second collection of 91 Napier grass genotypes was evaluated at the Embrapa Dairy Cattle experimental field, located in Brazil, and 5 cuttings were conducted between 2014–2016 in both wet and dry seasons. Rocha *et al*. (2019) described in detail these evaluations that were done in natural conditions. Nine genotypes (BA17, BA30, BA34, BA53, BA81, BA86, BA93, BA97, and Pioneiro (released cultivar)) were shared between these 2 trials. In both trials, the planting materials used were clonally propagated from original mother plants conserved in situ. Throughout the experimental period, the plants were harvested every 6–8 weeks, and no flowering was observed during the experimental period.

### Phenotyping of agronomic and feed quality traits

The following agronomic traits were measured for the trial carried out in Ethiopia: leaf length (LL, mm), leaf width (LW, mm), leaf-to-stem ratio (LSR), stem thickness (ST, mm), tiller number (TN), and biomass yield data (total fresh weight [TFW, g] and total dry weight (TDW, g) were collected as described previously in Habte *et al*. (2022). Water use efficiency (WUE) was also calculated by dividing the TDW per plant by the total volume of irrigated water applied to each plant during the dry season. Likewise in Brazil, plant height (PH, m), production of TFW (Mg ha^−1^), production of TDW (Mg ha^−1^), and DM concentration (%) were scored. Furthermore, 9 feed-quality nutritional traits including acid detergent fiber (ADF), neutral detergent fiber (NDF), lignin (LIG), cellulose (CEL), hemicellulose (HCEL), in vitro dry matter digestibility (IVDMD), ash (ASH), and nitrogen content (NIT) were also scored in Brazil. The DM concentration recorded for agronomic traits was used as a common denominator for estimating of biomass digestibility. Further details can be found in Rocha *et al*. (2019).

### Phenotypic data analysis

Collected phenotypic values for each trait were checked for normal distribution and transformed, when needed, ahead of variance comparison using the bestNormalize R package (Peterson 2018). Phenotypic variability between genotypes was calculated with R statistical software (R Core Team R 2022) using the model:

E · R + E · R · B + G + G · E where E, R, B, and G denote environment, replicate, incomplete block, and genotype, respectively. Environment effects and replicate effects nested within the environment, both represented by (E·R), were taken as fixed. In contrast, the block effect nested within the replicate and environment and the genotype-by-environment interaction (G·E) were taken as random. The main genotype effect (G) was taken as random. Analysis of variance (ANOVA) and multiple comparison tests (LSD) were conducted at a probability level of 5%. Furthermore, phenotypic data were used to carry out hierarchical clustering and principal component analysis (PCA) using the Factoextra R package (Kassambara 2017). Correlation analysis was also carried out between all the variables measured in the field evaluation in Brazil.

### Sequenced worldwide Napier grass collection

A total of 450 Napier grass genotypes were sequenced and deposited as bioproject PRJEB73794: 61 from the ILRI genebank, 131 from Embrapa, 23 from the USDA, 6 from China (Lanzhou University), 118 from the Kenya Agricultural and Livestock Research Organization, and 2 released cultivars, namely Super Napier (G1) and Pioneiro (PION). In addition, 109 progeny plants (generated from seeds collected from 14 ILRI genotypes (mother plants) by open pollination were sequenced. The progenies were from open pollinated plants in the field and the pollen donor genotypes were unknown. All mother plants were represented by 6–10 progenies except for 1 mother plant (IL18438), which a single progeny represented. More information about these genotypes can be found in Supplementary Table 1: metadata.

### DNA extraction and sequencing

Young leaf tissue was collected from respective genotypes and subjected to isolation of genomic DNA following the procedure described in the Qiagen DNeasy Plant Mini kit (250) (Qiagen Inc., Valencia, CA, USA). Before library preparation, DNA quality was checked on 1% agarose gels, and DNA purity was checked using a Nanophotometer spectrophotometer (IMPLEN, CA, USA), and DNA concentration was measured using the Qubit DNA Assay Kit in a Qubit 2.0 Fluorometer (Life Technologies, CA, USA). High-quality DNA with a minimum of 50 ng/µL was used for Illumina WGS. The genotypes were sequenced using Illumina technology using paired-end 2 × 150 bp short-reads. A total of 4.92 Tb of data were generated, with an average sequencing depth of 15–20 × per sample. Library preparation and sequencing were conducted by Novogene (https://en.novogene.com).

### Read mapping, SNP calling, and filtering

The quality of raw reads was checked using the FastQC (Andrews 2010) and MultiQc tools (Ewels *et al*. 2016). Afterwards, raw reads were trimmed and filtered with the trimmomatic tool (Bolger *et al*. 2014) to remove Illumina Truseq adapter remnant sequences, as well as low-quality reads (with a quality score lower than 30). Curated reads were mapped against the Napier grass reference genome with the Burrows Wheller Aligner (BWA) (Li and Durbin 2009). The SAM files generated from the BWA step were converted into sorted BAM files using SAMtools (Li *et al*. 2009). The HaplotypeCaller tool, Genome Analysis Toolkit (GATK4.4), was used for the variant calling step with default parameters (McKenna *et al*. 2010). The generated vcf file, from the variant calling step, was filtered and pruned with BCFtools (v.1.9) (Li *et al*. 2009). The SNP filtering process retained biallelic and polymorphic loci with read depths between 10 and 300, mapping quality (GQ > 20), a minor allele frequency above 0.05, and missing data in less than 1% of the samples. After filtering, 1,068,685 SNPs were retained for downstream analysis.

### Genetic diversity and population structure

The population structure analysis tool, ADMIXTURE (v1.3.0) (Alexander and Lange 2009), was used to infer optimal cluster/subpopulations (*K*) and the proportion of ancestry among the 450 global Napier grass genotypes, with the filtered SNPs. Ten independent runs were carried out for maximum likelihood estimates of the ancestry subgroups (*K*) from 2 to 10. For each *K*, ADMIXTURE was run 20 times with varying random seeds. Afterwards, CLUMPP software (Jakobsson and Rosenberg 2007) was used to align up to 10 Q-matrices in the same cluster. The number of ancestors was determined according to the position of the minimum value, with an error rate obtained from the cross-validation (CV) score. A good value of *K* will exhibit a low CV error compared to other *K* values. Outputs from ADMIXTURE were collated using the R pophelper program (v.2.3.1) (Francis 2017), which compares the ancestral make-up of each predicted population.

Using genotypic data, PCA was performed to examine inter-population distribution using the SNPRelate (v. 4.0.2) (Zheng *et al*. 2012) and Plotly R packages (Sievert 2020). A phylogenetic tree was also constructed with the filtered, high-quality SNPs using identity by descent with the SNPRelate R package (v.4.0.2) (Zheng *et al*. 2012) and visualized with the interactive Tree Of Life (Letunic and Bork 2021).

### Genome-wide association study

A total of 174 genotypes in 2 independent populations, 90 from Embrapa and 84 from ILRI, were considered for the genome-wide association study (GWAS). These genotypes included 2 independent populations phenotyped in the field evaluation carried out in Brazil and Ethiopia. These trials were carried out at 2 different times, and different traits were measured in each experiment. For the field evaluation carried out in Brazil, the marker-trait association analysis was carried out separately for dry and wet seasons, for each of the 12 quantitative agronomic and feed-quality traits (PH, TFW, DM concentration,TDW, CEL, LIG, ADF, NDF, HCEL, IVDDM, NIT and ASH). In the experiment carried out in Ethiopia, the traits measured were PH, leaf length (LL), LW, LSR, ST, TDW, TFW, TN, and WUE and the groups were split into MWS and SWS dry season treatments.

Furthermore, GWAS was employed to investigate marker-trait associations (MTAs) for 3 agronomic traits; PH, production of green biomass (TFW), and production of dry biomass (TDW) assessed in both field evaluations conducted in Brazil and Ethiopia. The mean value for each trait was first indexed as low, medium and high based on quantile values for each trait per season, i.e. mean values less than the 1st quantile were labeled as low and mean values between the 1st and 3rd quantiles were labeled as medium and mean values above the 3rd quantile were labeled as high. Once recoded, the data from each country was merged per trait and for each season. The transformed data from the 9 genotypes, shared between the 2 evaluations showed some degree of inconsistency (probably due to GxE interactions), particularly for the dry season and in such cases data from Ethiopia was selected since frequent measurements, i.e. every 8 weeks, were taken during the trial in Ethiopia (Supplementary Table 1).

Prior to GWAS, the average values for all traits were normalized using the bestNormalize R package (Peterson 2018). GWAS was then performed on the average values, normalized values, and BLUE and BLUP predicted values. Different GWAS models were used to ensure detection of significant associations while accounting for population structure and relatedness. The analysis was conducted using the Genomic Association and Prediction Integrated Tool (GAPIT) version 3 software package within the R environment (Wang and Zhang 2021). We implemented the Fixed and random model Circulating Probability Unification (Liu *et al*. 2016 ), which improves power by iteratively testing markers while controlling for confounding effects. Bayesian-information and Linkage-disequilibrium Iteratively Nested Keyway model (Huang *et al*. 2019) enhances efficiently by replacing the kinship matrix with Bayesian information and linkage disequilibrium (LD)-based marker selection. The multiple-locus mixed linear model (Segura *et al*. 2021) iteratively incorporates multiple associated SNPs as cofactors, improving polygenic trait detection while accounting for population structure and relatedness. The distribution of observed vs. expected −log10(*P*) values was assessed using Quantile–Quantile (Q–Q) plots, which visualize deviations from the null hypothesis. Significant SNP-trait associations were identified based on the internal model selection criteria and multiple testing correction methods implemented in GAPIT, including adjustments for population structure and control of the genome-wide type I error rate. This approach ensured the statistical rigor and reliability of our GWAS results.

Regions of 0.04 Mbp surrounding highly significant SNPs, identified by multiple models and associated with multiple traits and/or treatment conditions, were blasted against protein databases, including Phytozome (Goodstein *et al*. 2012), to identify homologous genes or proteins with similar sequences and MTAs. A threshold of 80% identity was used to report putative homologous proteins.

## Results

### Phenotypic variability among Napier grass genotypes

Field evaluations in wet and dry seasons at the Embrapa Dairy Cattle in Brazil indicated significant differences between seasons, among some agronomic and feed-quality traits (Table 1). PH and TFW were significantly higher during the wet season whereas CEL and ash concentrations exhibited no seasonal variation. Furthermore, the interaction between genotypes and harvest cycle was insignificant for most traits, except PH, TFW, DM, and TDW. Also, the interaction of genotypes with the season was significant for most traits except TDW, HCEL content, and NIT, indicating that the performance of genotypes was differentially affected by season. The mean performance per accession for all traits is presented in Supplementary Table  1.

**Table 1.** Mean square from combined analysis of varianice (ANOVA) for 12 growth and forage biomass yield traits of Napier grass accessions evaluated for 2 years in Brazil.

Source of variation	df	PH	TFW	DM	TDW	CEL	LIG	ADF	NDF	HCEL	IVDDM	NIT	ASH
Rep	1	4.1***	1830**	0.02***	67ns	79.4***	0.01ns	17.6***	4.2ns	61***	148.3***	0.55***	1.8***
Acc	90	0.83***	2036***	0.008***	289***	7.8***	3.2***	14.2***	12.1***	4.7***	30.7***	0.037***	3.57***
Harvest	3	41.97***	23,713***	0.2***	4360***	1636***	187.62***	2735.3***	160.9***	1785.6***	2575.6**	0.37***	47.65
Season	1	238.19***	145,149***	0.02***	4360***	1.1ns	189.4***	96.5***	323.3***	66.5***	1581.6***	0.3***	0.03ns
Acc:Harvest	267	0.13 ***	349***	0.0018***	48***	3.2ns	0.85ns	5.3ns	4.3ns	2.3ns	7.9ns	0.007ns	0.98ns
Acc:Season	89	0.23***	224*	0.0035***	28ns	6.2***	1.67***	11.5***	8.1***	2.5ns	14.4***	0.01ns	1.82**
Error	442	0.14	172	0.001	23	3.6	0.76	5.8	4.1	2.1	9.1	0.01	1.08

*p < 0.05.

**p < 0.01.

***p < 0.001.

df, degrees of freedom; PH, plant height; TFW, total fresh weight; DM, dry matter concentration; TDW, total dry weight; CEL, cellulose; LIG, lignin; ADF, acid detergent fiber; NDF, neutral detergent fiber; HCEL, hemicellulose; IVDDM, in vitro dry matter digestibility; NIT, nitrogen; ASH, ash; Acc, accessions.

Among the 91 genotypes evaluated, the highest TFW was recorded for genotypes BAGCE2, BAGCE64, and BAGCE60. The highest biomass-yielding genotypes as TDW were similar, indicating a high correlation between TFW and TDW. Regarding NIT content, a key trait in feed quality, the genotypes BAGCE58, BAGCE82, and BAGCE1 were the top performers (Supplementary Table 2). Interestingly the genotype BAGCE82, with the highest NIT content, also showed a high mean TFW (64.8 Mg ha^−1^).

**Fig. 1.**
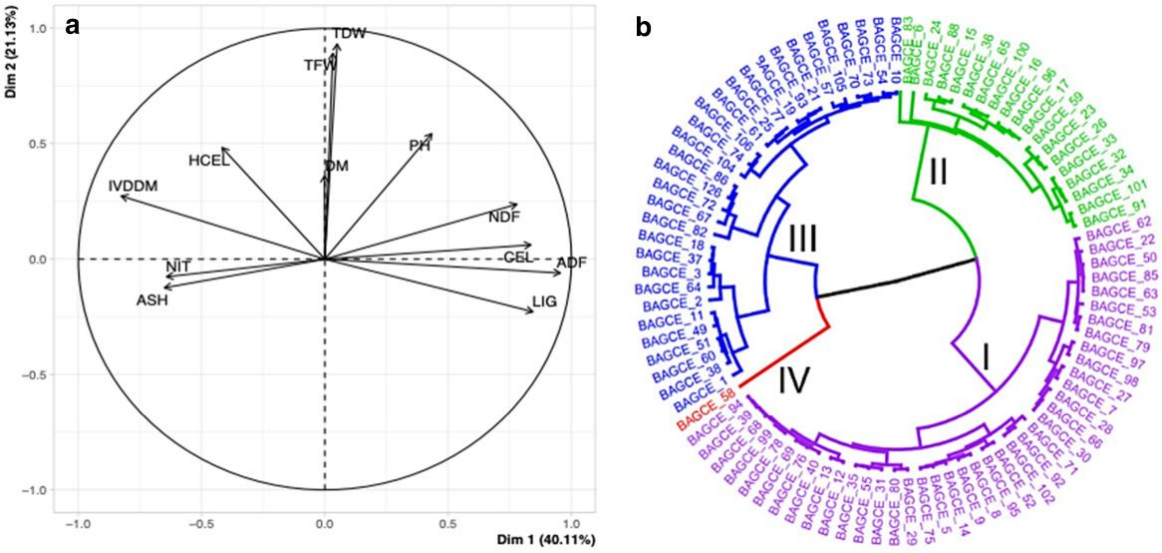
Principal component a) and cluster analysis b) of 91 Napier grass accessions evaluated in the Brazil trial, based on agro-morphological and nutritional traits. Accessions are grouped into four clusters shown in arbitrary colours for distinction.

The output from the field evaluation trial in Bishoftu, Ethiopia, has previously been reported (Habte *et al*. 2020). Among the shared genotypes, BAGCE53, 86 and 97 performed well regarding TFW in the trials in both Brazil and Ethiopia. PCA and clustering analyses were conducted among the subset of genotypes from Embrapa (Fig. 1). In this analysis divergence was observed based on growth, forage yield and nutritional quality traits. The PCA identified the first 3 components, explaining 77% of the cumulative variation (Supplementary Table 3). The first principal component (PC1) accounted for 40.1% of the total explained variance, PH (0.43), CEL (0.84), ADF (0.95), and NDF (0.78) were the main contributing traits for this component. Likewise, the second principal component (PC2) accounted for 21.1% of the total explained variance and PH (0.54), TFW (0.89), TDW (0.93), HCEL (0.48), and DM (0.36) were the main contributors for this component (Supplementary Table 3).

A PCA biplot shows the degree of correlation among measured traits, with those in the same dimension and a tight angle between vectors indicating a high and positive correlation (Fig. 1a). The strongest positive correlation was found between TDW and TFW. Furthermore, CEL and ADF were also highly and positively correlated. TFW, TDW, NDF and PH appeared in the same dimension with a positive correlation with each other, and CEL and ADF were negatively correlated with NIT and ash (Supplementary Fig. 1). Based on data from 2 seasons and 5 harvest cycles of mean values of 12 quantitative traits, the clustering analysis revealed 4 major clusters, cluster I, composed of 2 major sub-clusters, consisted of 40 genotypes; cluster II contained 3 major sub-clusters and consisted of 19 genotypes; cluster III contained 3 major sub-clusters and was composed of 31 genotypes; and the cluster IV exhibited a distinct genetic profile, forming an isolated group containing a single accession (Fig. 1b). Top ranking genotypes, in terms of TFW/TDW, such as BAGCE2, BAGCE60, and BAGCE64, were in cluster III. Interestingly, BAGCE58, which clustered in group IV, by itself, scored the lowest mean TFW and TDW (18.8 and 5.9 Mg, respectively) and the highest NIT content (0.66%).

### Genome-wide SNP discovery and their distribution across assembled chromosomes

Illumina 150-bp paired-end reads were generated from 450 Napier grass genotypes. The average sequencing depth was 15–20 × per accession. Nearly ∼170 million variants (SNPs and Indels) were generated and from these variants, ca. 1 M hard-filtered SNPs were mapped across the 14 assembled chromosomes of Napier grass (Supplementary Fig. 2). These markers were used for genetic diversity and marker-trait association analyses. The number of SNPs per chromosome was variable, with more SNPs mapped on the longest A01 and B01 chromosomes (Supplementary Table 4). The SNP density was similar for all chromosomes with 1 SNP detected for every 1,830 bases. We have generated a comprehensive Napier grass genome variation dataset, identifying numerous SNPs from diverse landraces, varieties, and progenies.

### Genetic variation and relationship

**Fig. 2.**
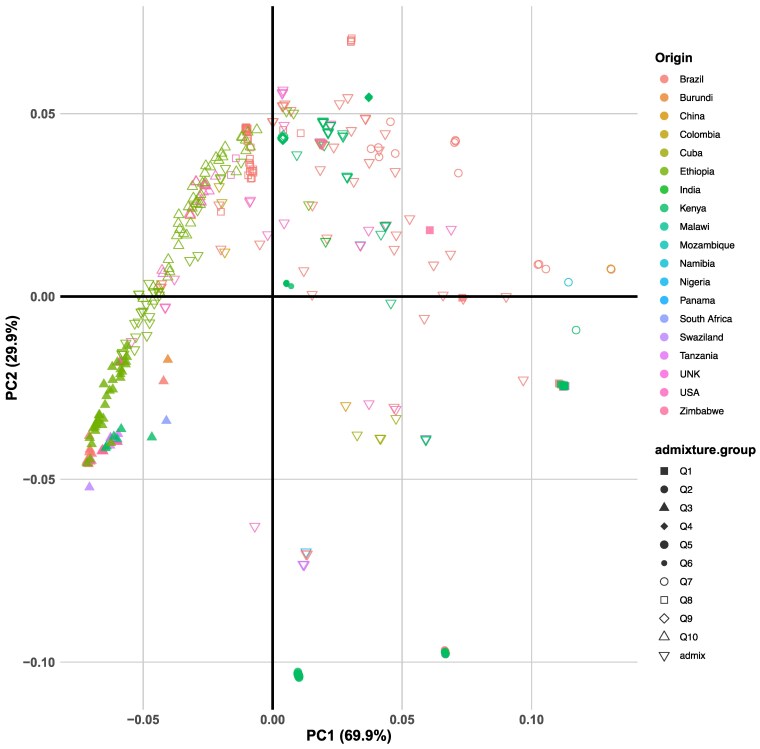
PCA of 450 Napier grass accessions based on approximately 1 million SNPs. The scatter plot shows the relationship between PC1 (explaining 69.9% of the variance) vs PC2 (explaining 29.9% of the variance). Data points color-coded by country of origin, and shapes represent distinct groups defined through ADMIXTURE analysis. Genotypes labeled “UNK” indicate unknown country of origin.

The PCA revealed a clear pattern of genetic structure, with 3 major clusters and a noticeable degree of aggregation based on the region of origin (Fig. 2). PC1 accounted for the largest proportion of genetic variation in the dataset (69.9%) while PC2 explained an additional 29.9%, together capturing nearly all of the total variation among the genotypes. Separation along PC1 primarily distinguished genotypes by admixture groups (Supplementary Table 5), with Q3 and Q10 forming distinct clusters apart from Q1 and Q7. In contrast, PC2 differentiated Q2 and Q5 from Q4 and Q8, which appeared as well-separated sub-clusters in the PCA space. The Q5 cluster consisted of mainly genotypes from Kenya, alongside 2 admixed genotypes sourced from ILRI while Q8 was predominantly composed of Brazilian accessions, with 2 samples representing USDA collection.

Interestingly, the 8 reported interspecific hybrid genotypes (Supplementary Table 6) did not form a distinct cluster, instead scattering across the PCA space. Similarly, genotypes sourced from ILRI were distributed among various clusters, reflecting their diverse global origins. Progeny genotypes were primarily grouped within Q10, although several appeared in other sub-clusters, mainly in Q3, suggesting varying degrees of relatedness or admixture. Notably, the admixed group (the largest, with 152 genotypes) encompassed accessions from germplasm banks in Ethiopia, China, and Brazil, as well as breeding lines and progeny. This group also included 5 of the 7 purple-colored genotypes (CN96273, CN96211, CN94131, CN93182, and CN93081), highlighting its genetic and phenotypic diversity.

### Population structure among global Napier grass genotypes

**Fig. 3.**
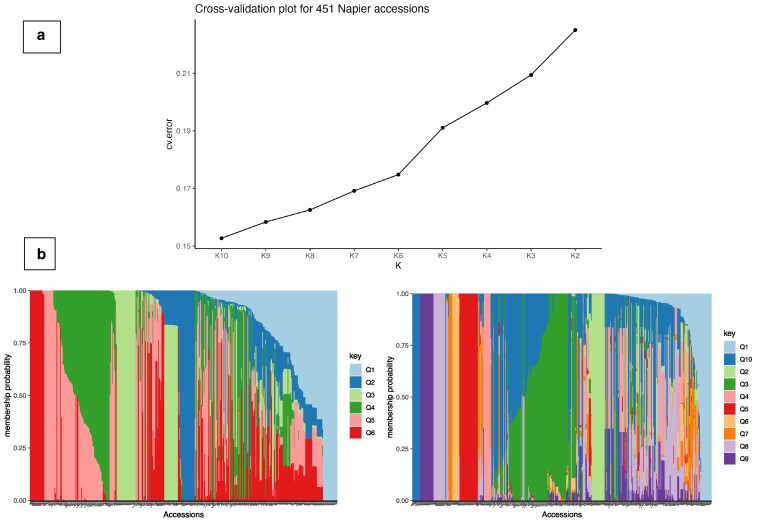
a) Cross validation error plot from ADMIXTURE analysis across *K* values ranging from 2 to 10, used to determine the optimal number of genetic clusters. b) Admixture bar plot showing population structure of 450 Napier grass accessions at *K* = 6 and *K* = 10.

**Fig. 4.**
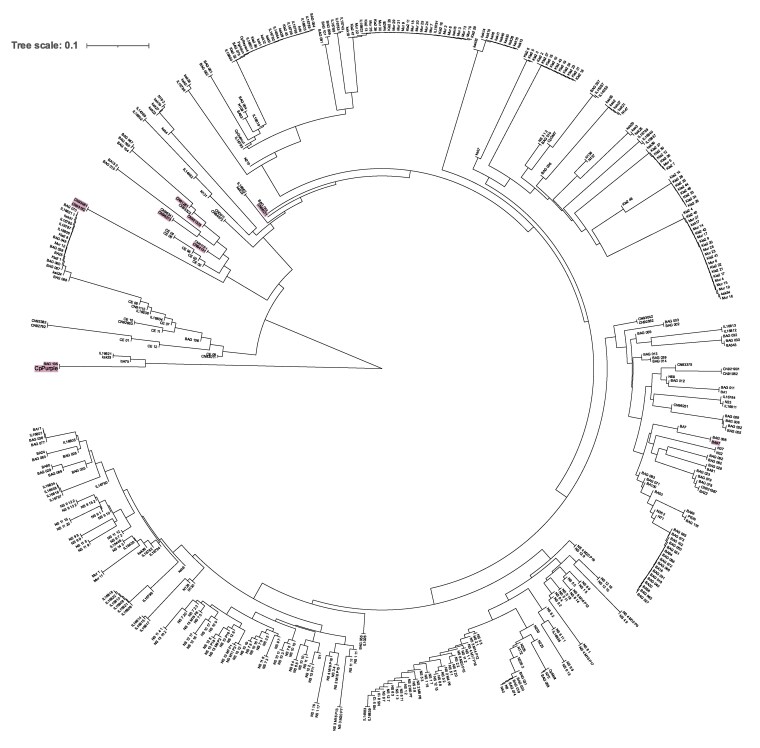
Phylogenetic relationships among 450 Napier grass accessions based on approximately 1 million filtered SNPs. Accessions with purple background exhibit purple leaf coloration, including the reference accession CpPurple which is among the purple-coloured accessions.

Population structure analysis divided the 450 genotypes into 10 subgroups according to CV errors (Fig. 3a). Clustering at *K* = 6 (Fig. 3b) which separated some genotypes from Embrapa (Brazil), Kenya, and ILRI from the rest. However, a high admixture was noticed within the overall collection. A similar trend was observed in the phylogenetic tree, where genotypes were distributed regardless of their region of origin (Fig. 4). For example, 2 genotypes of Chinese origin (cpReyan4 and PgJujun) clustered together with genotypes sourced from genebanks in Embrapa, Kenya, and ILRI. Interestingly, the reference genome CpPurple, a purple variety and all other purple varieties were clustered close to each other except BA97. Furthermore, CpPurple showed a high similarity with BAGCE105 (a purple accession from Brazil) indicating that these 2 could be related genotypes although currently grown on different continents. Progeny genotypes from ILRI showed a mixed trend in the phylogenetic tree; even those originating from the same mother plant did not cluster together. A high level of genetic similarity among Kenyan genotypes was observed indicating possible duplications in the Kenyan collection.

### Marker-trait associations

**Fig. 5.**
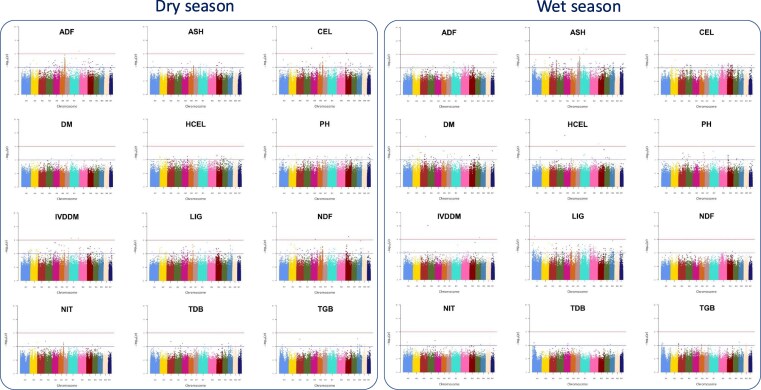
Significantly associated SNPs for the 12 quantitative traits evaluated during the dry a) and wet b) seasons in Brazil. Manhattan plots constructed with BLINK model. The GWAS analysis were performed for the following traits: ADF, ASH, CEL, DM concentration, HCEL, plant height (PH), IVDDM, LIG, NDF, NIT, TDB, and TGB. The horizontal lines represent the thresholds with *P*-value of; lower 0.05 [−log10(*P*) > 4] and upper; 0.01 [−log10(*P*) > 6], respectively. CEL, cellulose; ASH, ash; LIG, lignin; ADF, acid detergent fiber; DM, dry matter; NDF, neutral detergent fiber; HCEL, hemicellulose; IVDDM, in vitro dry matter digestibility; NIT, nitrogen; ASH, ash; TDB, total dry weight; TGB, total fresh weight

For the field evaluation carried out in Brazil, the marker-trait association analysis was carried out independently for dry and wet seasons for each of the 12 quantitative traits (Fig. 5). Significantly associated SNP loci [−log10(*P*) > 5], were identified for 10 traits scored in more than 1 association model for either of the seasons (Supplementary Fig. 3). Interestingly, significant QTL were recorded for some traits such as TFW and ADF [−log10(*P*) > 5] mainly in the dry season (Supplementary Table 7).

A GWAS was also carried out for the trial conducted in Ethiopia, and the results revealed interesting associations for the 9 traits scored. All traits significantly associated with QTL were identified under dry and wet seasons and also under 2 soil moisture conditions. A QTL significantly associated with ST was identified during the wet season and under both soil conditions in the dry season (Supplementary Table 8). Likewise, SNPs were identified to be significantly associated with TFW under both dry and wet seasons, in both soil conditions. For the binary trait, leaf color (green vs. purple), a total of 494 SNP loci were determined to be significantly associated [−log10(*P*) > 7.3] using 3 different GAPIT models (Supplementary Table 9). In general, a total of 207 SNP loci were significantly associated with other traits, excluding the leaf color, using 3 GAPIT models, for the trial carried out in Ethiopia (Supplementary Fig. 4). Forty-seven of those marker trait associations (MTAs) were significantly associated with more than 1 trait and were also significant in more than1 GAPIT model. Three traits, PH, TFW and TDW were measured in both field trials in Ethiopia and Brazil, and the combined data were used to carry out a GWAS analysis that led to the identification of additional QTL (Supp. Fig. 5). Interestingly, significant QTL were identified for all 3 traits at a higher threshold [−log10(*P*) > 7.3] in the dry season. In contrast, a significant QTL was only identified for TDW in the wet season.

From the significantly associated QTLs, 13 were associated with multiple phenotypic and feed quality traits with more than 1 GAPIT model. The search for those significant MTAs has revealed sequence similarity with proteins of various functions in the Phytozome database. For instance, a region around a QTL associated with TDW and TFW showed sequence similarity with a Gag-Pol-related retrotransposon. Similarly, another QTL significantly associated with traits such as ADF, IVDMD, and LIG showed similarity with a WDSAM1 protein (Supplementary Table 10). Additionally, a QTL highly correlated with leaf color exhibited similarity with the Zinc-finger domain of a monoamine-oxidase A repressor R1 and carotenoid synthesis regulator regions.

## Discussion

Tropical forages, compared to temperate counterparts like perennial ryegrass and alfalfa, remain under-researched. Consequently, most small-scale farmers, especially in Africa, rely on landraces, which may lack adequate adaptation to the current and future climate conditions (Simeão *et al*. 2021). Among tropical grasses, Napier grass (also known as Elephant grass) is widely grown in SSA due to its high biomass, resilience to heat and water scarcity, and ability to regrow for up to 6 harvests annually (Kamau 2007; Habte *et al*. 2022).

### Field evaluation of Napier grass genotypes

The evaluation of Napier grass genotypes from ILRI (Ethiopia) and Embrapa (Brazil) highlighted important phenotypic performance and diversity. These evaluations were essential for identifying promising candidates for breeding, with a focus on improving biomass, drought resilience, and feed quality traits such as high crude protein and low LIG. Consistent with previous findings (FAO 2010; Njuki and Sanginga 2013; Lamb *et al*. 2018), traits like PH, TFW, and TDW were higher during the wet season compared to the dry season. In contrast, feed quality traits like CEL and ash content remained stable across seasons. Notably, genotypes such as BAGCE2, BAGCE64, and BAGCE60 showed the highest TFW and TDW, performing well in both dry and wet seasons, suggesting strong genetic potential. BAGCE30, which was shared between the trials in Brazil and Ethiopia, demonstrated resilience to drought, producing consistently high biomass in both countries, further supporting its potential for performance across diverse environments (Habte *et al*. 2022).

Feed quality traits such as crude protein (calculated as % nitrogen × 6.25) and LIG contents are key forage traits. In a trial conducted in Brazil, the highest NIT was recorded for accession BAGCE58, although it ranked the lowest for TFW and TDW (Supplementary Table 2). Positive correlations were observed between traits, such as TFW and TDW, CEL and ADF (Supplementary Fig. 1), consistent with findings by Habte *et al*. (2022). Conversely, CEL and ADF were negatively correlated with NIT and ASH.

Napier grass is a promising bioenergy crop due to its high CEL, HCEL, and LIG contents. In this study, CEL content across genotypes ranged from 36.9 to 43.1%, with BAGCE83, BAGCE6, and BAGCE59 having the highest levels, making them suitable for ethanol production. Pioneiro, a Brazilian livestock forage cultivar, had the lowest CEL content, along with BAGCE104, BAGCE106, and BAGCE62, suggesting potential for improving digestibility through breeding. Genotypes clustered into 4 groups based on agro-morphological and feed quality traits. Cluster IV, containing BAGCE58, had the lowest TFW and TDW but the highest NIT content, which could be valuable for nitrogen production in breeding programs. High-yielding genotypes like BAGCE2, BAGCE60, and BAGCE64 were in Cluster III, showing strong potential for future breeding.

### Genomic tools for Napier grass

Previous genotyping by sequencing (GBS) studies on Napier grass identified around 100 K SNP markers, with lower SNP density compared to the current study (Paudel *et al*. 2018; Muktar *et al*. 2019,^2022^, ^2023^). WGS of 450 global Napier grass genotypes, mostly landraces, generated over 100 million variants, revealing significant genetic diversity. In the present study, an average of 1 SNP was detected every 1803 bases across all chromosomes (Supplementary Table 4). After a complex filtering, nearly a million SNPs were retained, evenly distributed along the 14 Napier grass chromosomes. These genome-wide SNPs can be used as a DNA fingerprinting tool in the germplasm bank collections and to verify the trueness-to-type of cultivars. As an allotetraploid (2*n* = 4× = 28, A′A′BB sub-genomes), Napier grass shares high homology with the pearl millet A genome (*Cenchrus americanus*, 2*n* = 2× = 14, AA), suggesting that genomic tools developed here could be useful for both Napier grass and pearl millet improvement or hybrid development (Gupta and Mhere 1997). The WGS approach applied in this study has the potential to generate more SSR markers compared to the previous GBS-based approach (Paudel *et al*. 2018).

### Inter-population structure and phylogeny among global Napier grass genotypes

PCA of filtered SNPs revealed 3 major clusters (Fig. 2), but these did not align with region of origin. This may be due to clonal propagation of Napier grass and limited genetic selection in the samples, which were sourced from 18 different countries. A similar finding was reported by Muktar *et al*. (2023) using GBS genotyping and Wanjala *et al*. (2013) with AFLP markers, where genotypes did not cluster by region of origin. However, some genotypes, such as Q4 and Q6, representing only Kenyan samples from different districts (Kiambu and Murang’a), showed regional aggregation. This likely reflects the historical and ongoing exchange of root splits through Kenya's informal seed system as noted by Muktar *et al*. (2023). An interesting finding from the PCA was that progenies from 14 mother plants grouped separately and did not show a distinct profile reflecting their sexual origin. A similar result was reported by Muktar *et al*. (2023) using GBS genotyping, where progenies did not cluster with their respective mother plants. Several ILRI genotypes aggregated closer to the Embrapa elite breeding lines, which contributed to cultivars like BRS Capiacu and BRS Kurumi (Pereira *et al*. 2017), suggesting their genetic potential for cultivar improvement. This study also included 8 hybrids (*Cenchrus purpureus* × *Cenchrus americanus*), but they did not cluster independently, indicating a possible error in their acquisition or management. However, further taxonomic and/or cytology characterization is needed to confirm their hybrid status.

Population structure analysis was conducted to better understand the relationships among genotypes, landraces, breeding lines, and progeny plants. The analysis divided the 450 genotypes into 10 subgroups based on CV error values from an ADMIXTURE analysis (Fig. 3b), which separated some of the Embrapa genotypes from ILRI genebank materials (Supplementary Table 5). A similar trend was reported by Muktar *et al*. (2023), who used GBS to distinguish Embrapa and ILRI collections. Additionally, Negawo *et al*. (2018) observed 2 sub-populations in nearly 2 hundred Napier grass genotypes from the same collections using SSR markers. This finding suggests that the ILRI and Embrapa collections represent 2 independent gene pools with slight admixture, indicating that heterotic breeding for desirable traits would be effective. Interestingly, most progenies were distributed across different sub-clusters, consistent with the PCA analysis. This pattern may be due to gene recombination during hybridization, a similar unorthodox clustering of progenies was also noted by Muktar *et al*. (2023).

A phylogenetic tree of the 450 genotypes confirmed previous findings, showing no clustering based on region of origin. It revealed 2 main clusters: 1 cluster with 5 genotypes and the other containing all remaining samples. The first cluster included CpPurple (the reference genome), 2 Embrapa genotypes and 2 accessions, from Kenya and from ILRI. Notably, CpPurple (a purple variety) showed high genetic similarity to BAGCE105, another purple genotype, despite being sourced from China. Similarly, Pioneiro and BAGCE116 clustered closely, even though BAGCE116 was selected from a different elephant grass population. BAGCE116 has distinctive yellow-green striped leaves, suggesting it may be a mutant variant of the Pioneiro cultivar, though further validation is needed.

As seen in the PCA, hybrid-labeled genotypes (IL16835, IL16837, IL16834, IL16838, IL15357, IL16840, and IL14982) did not cluster together in the phylogeny tree, failing to reflect a distinct hybrid profile. This aligns with findings by Muktar *et al*. (2019). Additionally, a high level of genetic similarity or possible duplication was observed among Kenyan-sourced genotypes. Muktar *et al*. (2023) similarly reported low genetic diversity among Kenyan genotypes, despite their collections from different districts.

Napier grass exhibits self-incompatibility (Martel *et al*. 1997; Hanna *et al*. 2004) and is obligate outcrossing in nature (Yan *et al*. 2021). The phylogenetic tree generated from this study can aid in selecting distantly related parents to develop hybrid varieties. Hybrid breeding has been effective in temperate forages like ryegrass (Foster 1971; Pembleton *et al*. 2013) and could similarly benefit Napier grass, its vegetative propagation allows for target traits to be fixed at the F_1_ stage. Overall, the phylogeny aligns with PCA and ADMIXTURE analyses, confirming the genetic diversity in this collection without clear clustering by region of origin.

### Genome-wide association studies identified key QTL in two different collections

Despite its resilience to various biotic and abiotic stresses, Napier grass production faces challenges from head smut and stunt diseases, recurrent droughts, and feed quality issues. Developing high-yielding, nutritious, and stress-resilient varieties is essential for improving animal performance, particularly in the SSA region. However, field characterization is time consuming and labor-intensive, mainly due to its perennial nature (Habte *et al*. 2020), large size (unsuitable for greenhouse studies; Rengsirikul *et al*. 2013), and obligate outcrossing reproduction (Martel *et al*. 1997).

Molecular markers are crucial for accelerating breeding and reducing resource use. Studies on wheat have shown that genomic-assisted selection significantly improves yield compared to traditional phenotypic selection (Tessema *et al*. 2020). While only a few GWAS have been conducted on Napier grass (Muktar *et al*. 2019; Rocha *et al*. 2019; Muktar *et al*. 2022), the high-density genome-wide SNP markers reported here will improve identification of markers linked to key traits, such as total fresh and dry weight (TFW, TDW) and WUE. In this study, a GWAS was performed on the 2 independent collections, revealing important marker trait associations (MTAs). For the field evaluation in Brazil, SNPs [−log10(*P*) > 5] were significantly associated with all measured traits in at least 1 GAPIT model across both seasons (Fig. 5). Overall, 318 SNPs were linked to 12 traits under both dry and wet conditions (Supplementary Table 7). These MTAs could aid future Napier grass breeding efforts in Brazil and beyond. Additionally, the associated SNPs may help identify genes underpinning important traits, providing targets for gene editing, a key tool for improving animal productivity in the tropics (Camargo and Pereira 2022). However, we acknowledge the limitations of our sample size and future work incorporating larger and more diverse panels will be valuable for further validating these results.

In the Ethiopian trial, a higher number of QTL were identified for key agronomic traits such as TFW, TDW, and TN, all critical for Napier grass improvement. Significant associations were observed across dry and wet conditions and under moderate (MWS) and severe (SWS) soil moisture stress (Supplementary Table 8). Muktar *et al*. (2022) previously reported QTL linked to yield, WUE, and feed quality traits, with significant MTAs for TDW under dry and SWS conditions on Chr5, Chr9, and Chr13. This study identified additional MTAs across most of the Napier grass chromosomes (Supplementary Table 8).

ST showed significant QTL [−log10(*P*) > 5] during the wet season harvests and under both MWS and SWS in the dry season (Supplementary Fig. 4). Notably, GWAS identified 494 highly associated SNPs [−log10(*P*) > 7.3] for the binary leaf color trait (green vs. purples), distributed across all chromosomes (Supplementary Table 9). Muktar *et al*. (2022) also reported GBS markers linked to purple leaf color, but this study identified a greater number of SNPs across both sub-genomes. Since purple pigmentation in Napier grass results from high anthocyanins content, which has potential health benefits for both humans and animals (Kruger *et al*. 2014; Yao *et al*. 2016), these MTAs could be valuable for feed quality improvement. Three traits, PH, TFW, and TDW were measured in both Ethiopian and Brazilian field trials. The combined analysis revealed QTLs that were consistent across environments. Interestingly, during dry-season treatments, significant QTL were identified for all 3 traits at a higher threshold [−log10(*P*) > 7.3], while in the wet season, significant QTL were detected only for TDW (Supplementary Fig. 5). One of the SNPs significantly associated with leaf color (A01_63825491) was located in a region syntenic with homologs encoding protein-serine/threonine kinases (Supplementary Table 10). These proteins act as central regulators, processing environmental and external cues to influence gene expression, metabolism, growth, development, fertilization, and immunity (Hardie 1999; Jose *et al*. 2020; Liu *et al*. 2024).

Another SNP (A01_ 63717178) linked to leaf color was found in the region associated with genes involved in the carotenoid biosynthesis pathway, which plays a key role in producing photosynthetic pigments, stress hormones, and protective compounds in grass species like rice (Shumskaya and Wurtzel 2013; Stanley and Yuan 2019). Carotenoids also have antioxidant properties, helping to reduce diseases incidence in both plants and animals (Abdelali and Zakir 2016). The SNP markers identified in this study could aid in developing nutritionally enhanced Napier grass cultivars.

### Conclusions

Limited access to high quality forages significantly impacts livestock performance in SSA. Indigenous species like Napier grass, which is familiar to smallholder farmers, require low inputs and adapt well to various agro-ecologies and production systems, are recommended. Our results reveal genomic differences and marker trait associations in global Napier grass genotypes, likely due to adaptation to diverse environments and breeding. We believe that the genomic tools developed, including the diversity profile and identified QTL, alongside recently available reference genomes, will promote the use of molecular markers in Napier grass improvement. These resources are also vital for managing genetic diversity and advancing conservation programs both in situ on farms and ex situ in genebanks.

## Supplementary Material

jkaf113_Supplementary_Data

## Data Availability

All data generated and analyzed in this study are publicly available. The raw sequencing reads have been deposited in the European Nucleotide Archive (ENA) under the accession number PRJEB73794: https://www.ebi.ac.uk/ena/browser/view/PRJEB73794. The complete SNP dataset is accessible via the European Variation Archive (EVA) under the accession number PRJEB88573: https://www.ebi.ac.uk/eva/?eva-study=PRJEB88573. Supplemental material available at G3 online.
