# Supplementary material for: Whole-genome resequencing of a global collection of Napier grass (Cenchrus purpureus) to explore global population structure and QTL governing yield and feed quality traits
Source: G3 (Bethesda). 2025 May 23;15(7):jkaf113. doi: 10.1093/g3journal/jkaf113 (PMC12239605; doi:10.1093/g3journal/jkaf113)
Supplement: jkaf113_Supplementary_Data [file jkaf113_supplementary_data.zip › Supplemental_Figures_G3-2025-405875.docx]

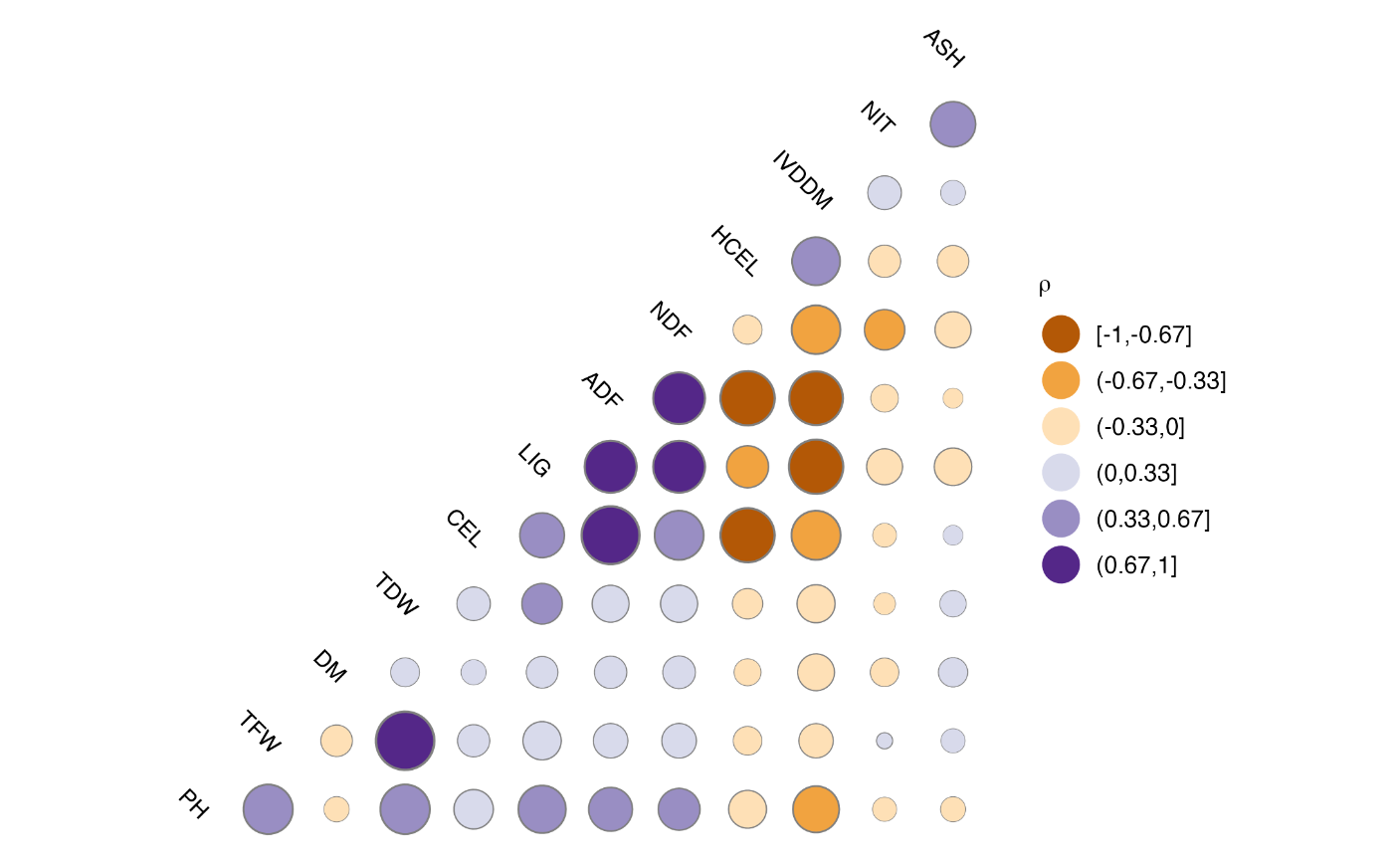


**Supp. Fig 1**. Correlation matrix plot of 12 quantitative traits measured in field trial in Brazil


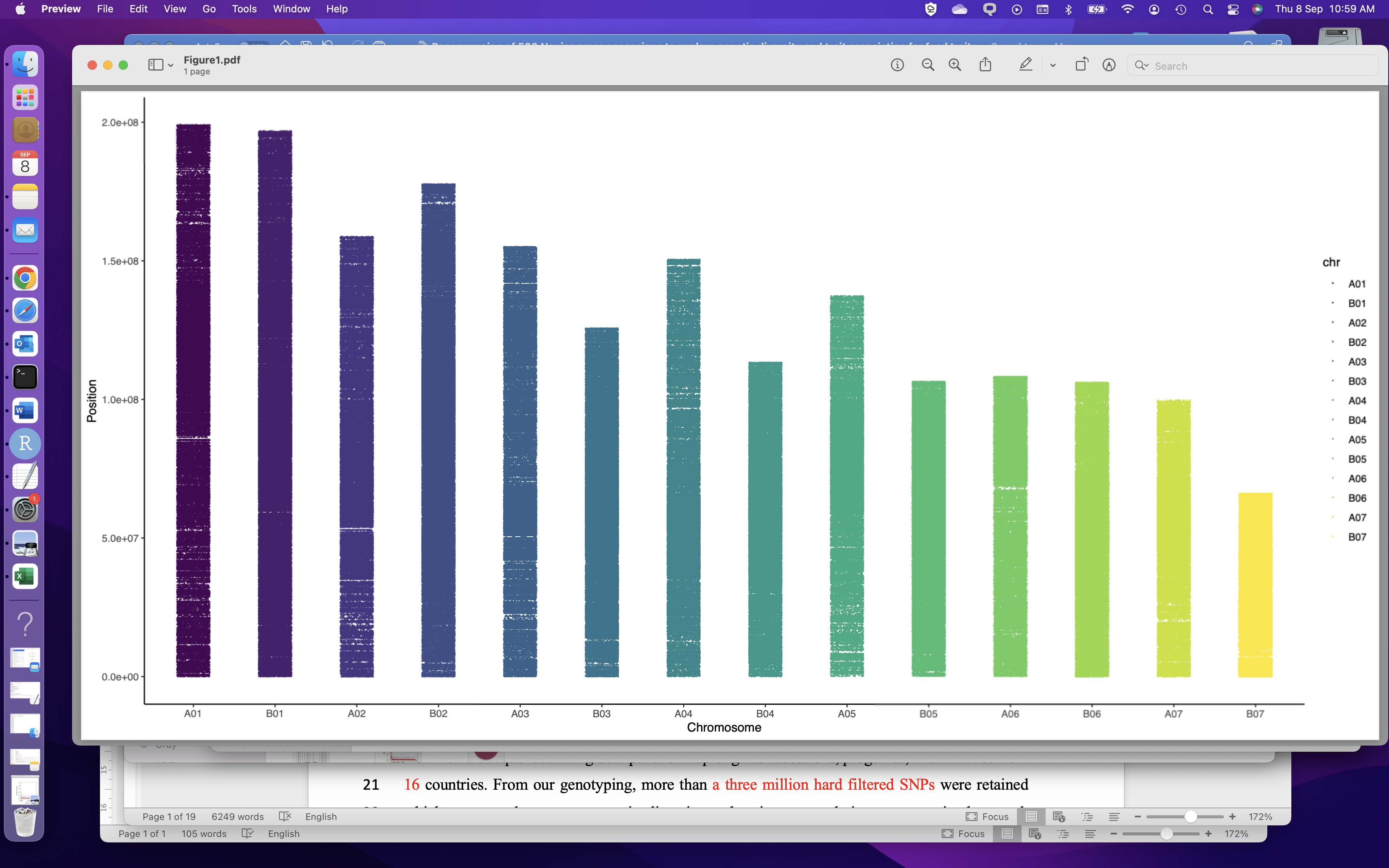


**Supp. Fig 2**. Distribution of a million SNPs among 14 Napier grass chromosomes


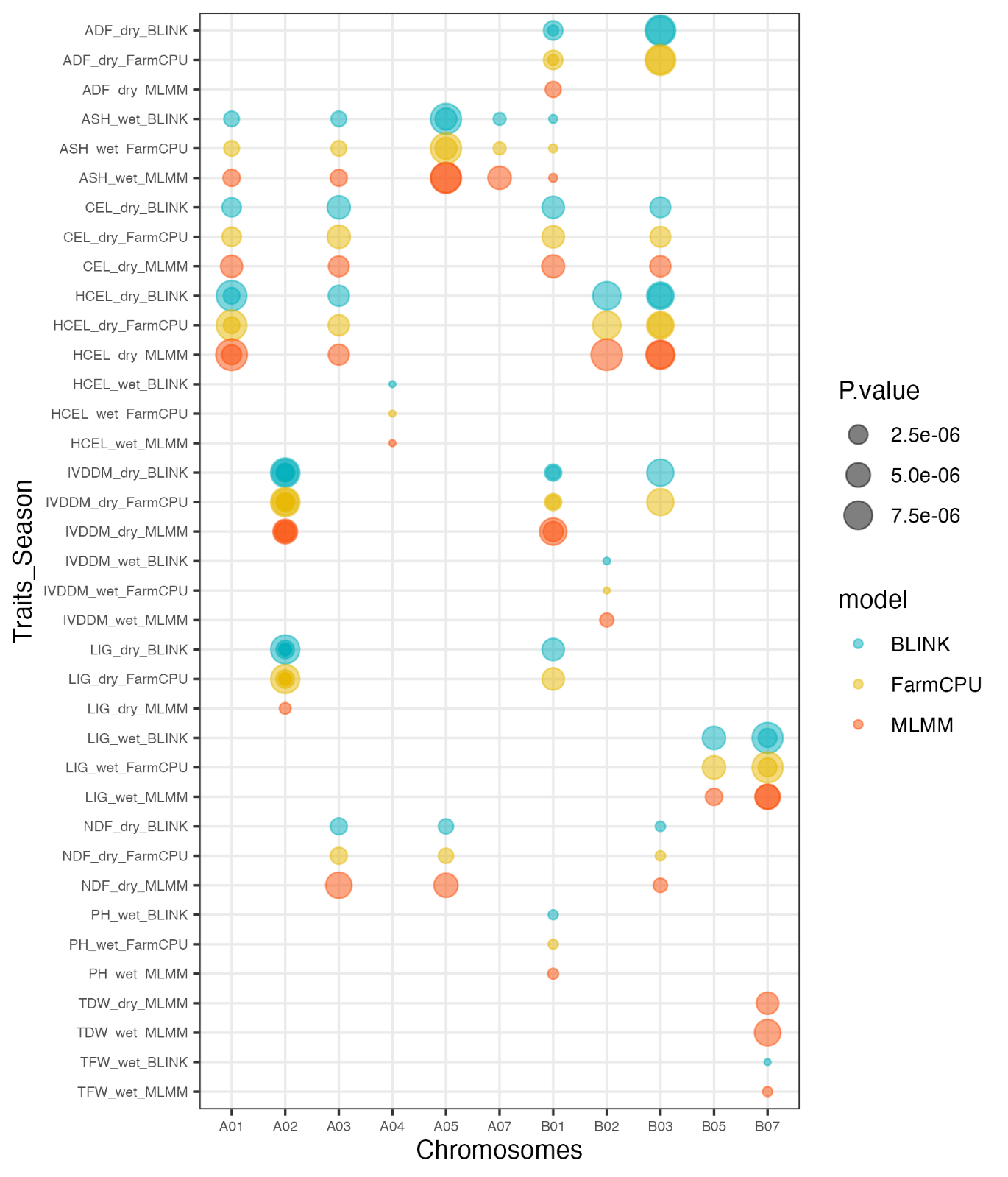


**Supp**. **Fig**. **3**. Bubble plot highlighting significantly associated SNPs (5 < −log10(p)) for 12 measured traits (dry and wet season) for the field trial in Brazil, with three GAPIT models


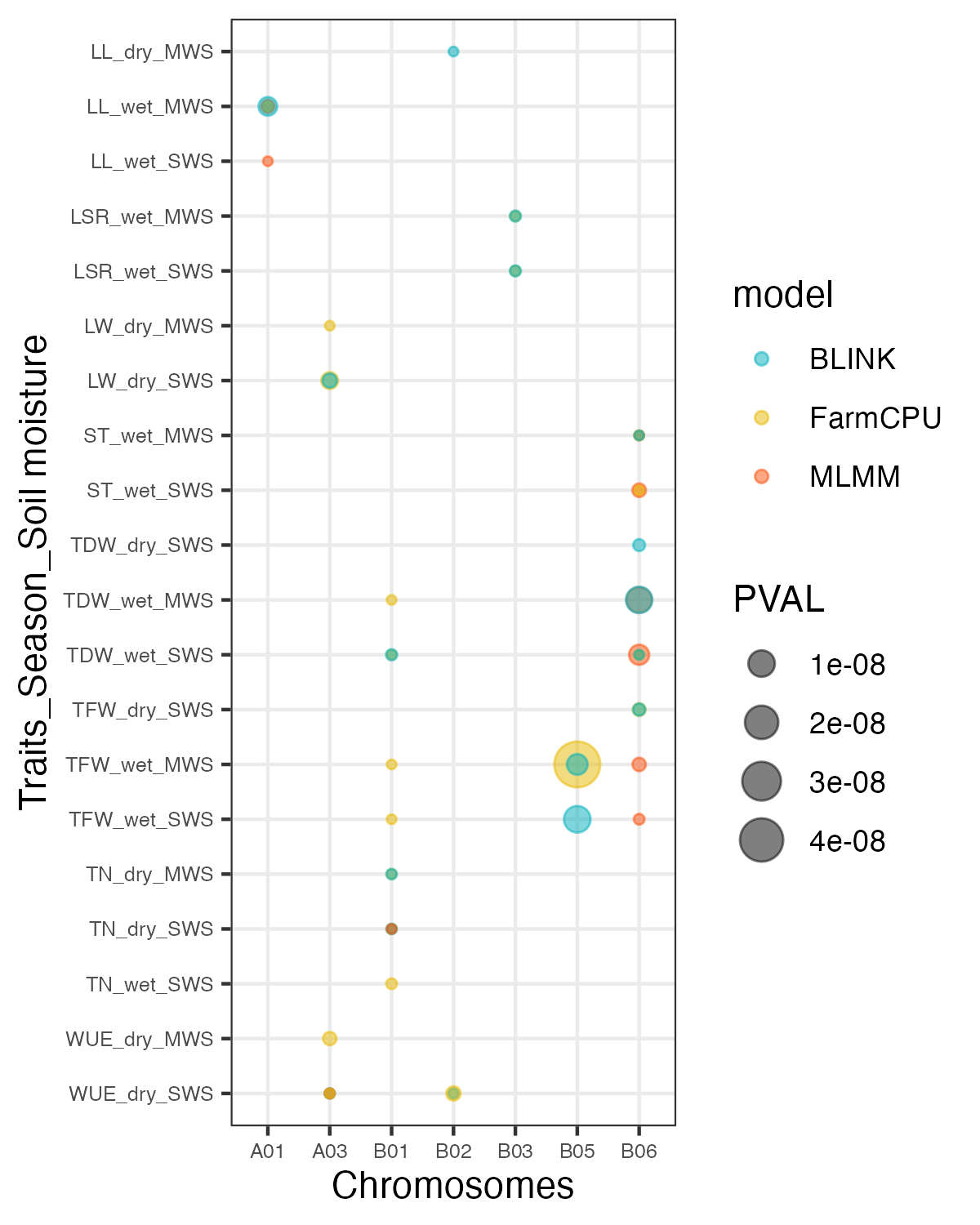


**Supp**. **Fig**. **4**. Bubble plot highlighting significantly associated SNPs (8 < −log10(p)) for 12 measured traits (dry and wet season) for the field trial in Ethiopia, with three GAPIT models


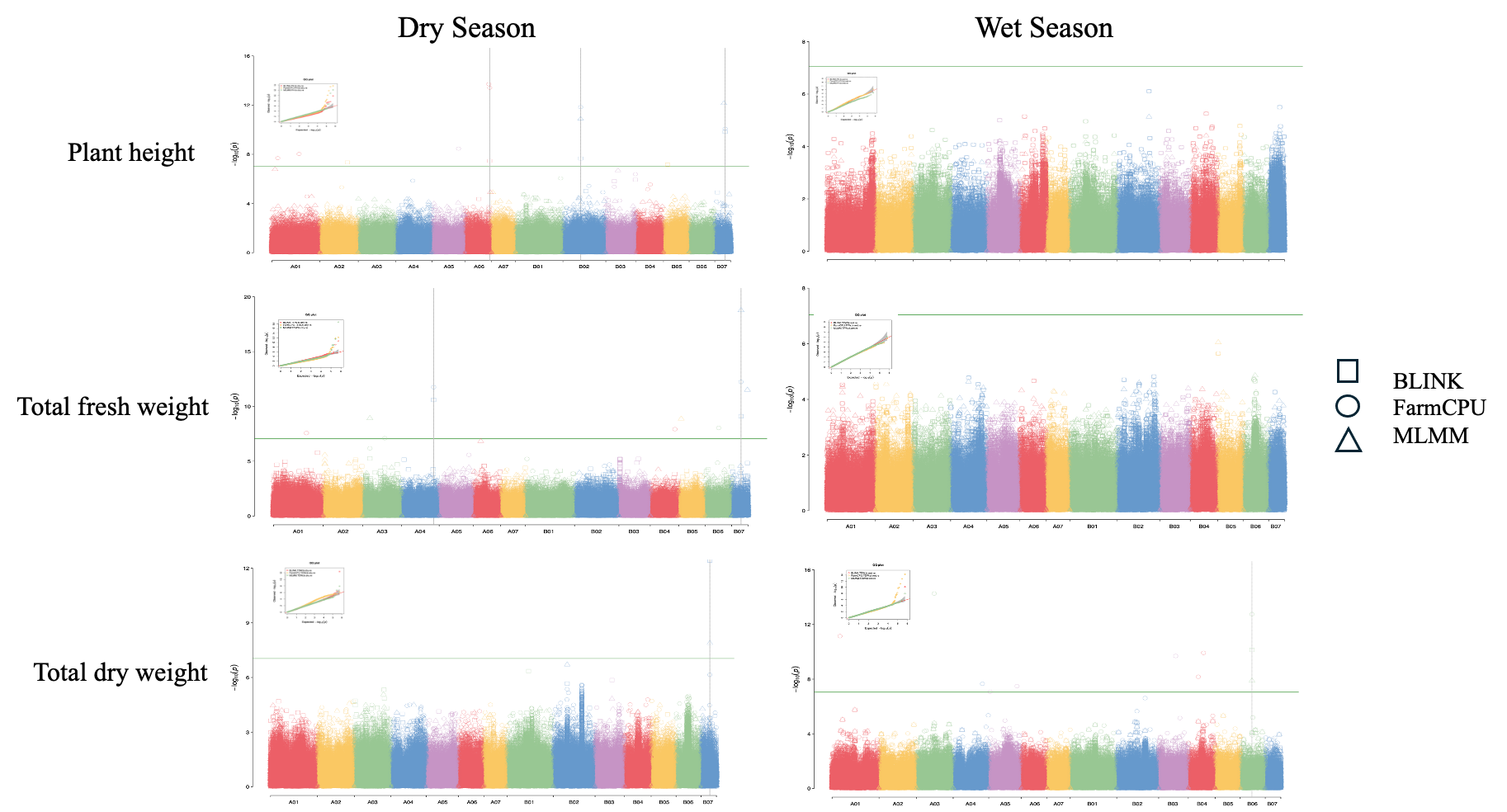


**Supp. Fig. 5.** Manhattan plot of a genome-wide association study on three traits, plant height, total fresh weight and total dry weight with three GAPIT models. The genome-wide significance level is set at 7 × 10−8 and plotted as the dotted line. The most significant SNPs are above the aforementioned threshold. Quantile-quantile (QQ) plot for each analysis as also shown in the Manhattan plot. The different shapes correlated with three GAPIT models used
